# Role of the Oxytocin Receptor Expressed in the Rostral Medullary Raphe in Thermoregulation During Cold Conditions

**DOI:** 10.3389/fendo.2015.00180

**Published:** 2015-11-25

**Authors:** Yoshiyuki Kasahara, Yuko Tateishi, Yuichi Hiraoka, Ayano Otsuka, Hiroaki Mizukami, Keiya Ozawa, Keisuke Sato, Shizu Hidema, Katsuhiko Nishimori

**Affiliations:** ^1^Laboratory of Molecular Biology, Department of Molecular and Cell Biology, Graduate School of Agricultural Science, Tohoku University, Miyagi, Japan; ^2^Department of Disaster Psychiatry, International Research Institute of Disaster Science, Tohoku University, Miyagi, Japan; ^3^Division of Genetic Therapeutics, Centre for Molecular Medicine, Jichi Medical University, Tochigi, Japan

**Keywords:** oxytocin, oxytocin receptor, rostral medullary raphe, body thermoregulation, energy homeostasis

## Abstract

Recent papers have reported that oxytocin (Oxt) and the oxytocin receptor (Oxtr) may be involved in the regulation of food intake in mammals. We therefore suspected the Oxt/Oxtr system to be involved in energy homeostasis. In previous studies, we found a tendency toward obesity in *Oxtr*-deficient (*Oxtr*^−/−^) mice, as well as impaired thermoregulation when these mice were exposed to cold conditions. In the present study, we observed the expression of Oxtr in the rostral medullary raphe (RMR), the brain region known to control thermogenesis in brown adipose tissue (BAT). Through immunohistochemistry, we detected neurons expressing Oxtr and c-Fos in the RMR of mice exposed to cold conditions. Up to 40% of Oxtr-positive neurons in RMR were classified as glutamatergic neurons, as shown by immunostaining using anti-VGLUT3 antibody. In addition, mice with exclusive expression of Oxtr in the RMR were generated by injecting an AAV-Oxtr vector into the RMR region of *Oxtr*^−/−^ mice. We confirmed the recovery of thermoregulatory ability in the manipulated mice during exposure to cold conditions. Moreover, mice with RMR-specific expression of Oxtr lost the typical morphological change in BAT observed in *Oxtr*^−/−^ mice. Additionally, increased expression of the β3-adrenergic receptor gene, *Adrb3*, was observed in BAT. These results are the first to show the critical role of RMR Oxtr expression in thermoregulation during cold conditions.

## Introduction

Oxytocin (Oxt) is a member of the nonapeptide hormone family and is synthesized in the neurons of the supraoptic nucleus (SON) and paraventricular nucleus (PVN) of the hypothalamus (1, 2). Magnocellular Oxt neurons in these nuclei project their axons to the posterior lobe of the pituitary gland, where they secrete Oxt into blood vessels (3, 4).

The Oxytocin receptor (Oxtr) is widely expressed in the reproductive tract (i.e., uterus, mammary gland, ovary, testis, and prostate), brain, kidneys, and many other organs in mammals (1, 2, 5–9). The Oxt/Oxtr system is involved in wide variety of physiological functions, and is best known for its role in lactation and parturition. Furthermore, recent papers have reported on the role of Oxt in the modulation of the estrous cycle, penile erection, ejaculation, and bone formation (1, 6–11). The central functions of Oxt/Oxtr have been studied in addition to these peripheral roles, and it has been reported that the Oxt/Oxtr system is important for social behavior, including sexual behavior, maternal behavior, affiliation, and social memory (5, 7, 8). Oxt has also been implicated in the regulation of energy homeostasis, including food intake and thermoregulation (12–15). Central administration of Oxt with an Oxt agonist decreases food intake in rats, and nesfatin (a satiety hormone) increases Oxt secretion (12, 16). These reports indicate that Oxt may act to inhibit food intake. Moreover, previous reports have indicated that central administration of Oxt induces hyperthermia (17–19). This is supported by the existence of a group of Oxt neurons in the hypothalamus that project polysynaptically to brown adipose tissue (BAT) (20), which plays an important role in adaptive thermogenesis (21).

There are two types of thermogenesis in mammals: shivering thermogenesis caused by the movement of skeletal muscle, and non-shivering thermogenesis attributed to the essential function of BAT. Cold exposure induces shivering and sympathetic nerve activation, which innervates BAT (21). Several studies have reported an abnormal swelled morphology of adipocytes in the BAT of mutant mice with dysfunctional thermoregulation (22–24).

The central nervous system modulates thermogenesis under cold conditions. By analyzing retrograde neuron tracers and c-Fos expression central control of thermogenesis was shown to be regulated by the feed-forward reflex pathway composed of the lateral parabrachial, median preoptic, dorsomedial hypothalamic, medullary raphe, and spinal intermediolateral nuclei (IML) (25, 26). Rostral medullary raphe (RMR) neurons at the end point of this pathway play a key role in thermogenesis via sympathetic premotor neurons (SPNs), which regulate sympathetic BAT thermogenesis (25, 27, 28). The raphe pallidus (RPa) and raphe magnus nuclei (RMg) are representative nuclei that are part of the RMR and contain specific neurons that control thermogenesis in BAT. Activation or inhibition of these neurons results in the induction or suppression of BAT activity (25). The possible regulation of BAT activity by neurons projecting from the RMR to the BAT has been implicated by the detection of fluorescent protein signal in the RMR following the injection of pseudorabies virus (as a retrograde tracer) into the interscapular BAT (25). In addition to non-shivering thermogenesis, the previous study has reported that shivering thermogenesis is also controlled by the same brain region (29).

Anatomical studies indicate that neurons in the RMR, which project to the IML and modulate BAT SPNs, express either vesicular glutamate transporter 3 (vGluT3) or serotonin and its synthetic enzyme, tryptophan hydroxylase (25). These neurons express c-Fos in response to exposure to cold conditions or an intracerebroventricular injection of prostaglandin E2. A nanoinjection of a glutamate receptor agonist into the T4 IML region can induce activation of BAT SPNs, and inhibition of glutamate receptors in this region suppresses thermogenesis in BAT (25). Furthermore, a nanoinjection of serotonin into the T4 IML can also activate BAT SPNs and BAT thermogenesis, and serotonin was shown to have the additional ability of inducing increased glutamine receptor-mediated potentiation in BAT SPNs. Similar to glutamate receptor antagonism, serotonin receptor antagonism in the T3-T6 IML region also prevented the evoked stimulation of BAT SPNs in response to cold conditions (30).

In previous reports, we have suggested that both *Oxt*-null and *Oxtr*-null mice show aberrant thermoregulatory function (13–15). In addition, *Oxtr*-deficient (*Oxtr*^−/−^) mice have a morphological defect in BAT cells and exhibit obesity (13, 15). We hypothesized that the central Oxt/Oxtr system is involved in thermoregulation, including BAT. Our previous study suggested that Oxtr in the dorsomedial hypothalamus and/or ventromedial hypothalamus is important for thermoregulation, but the function of Oxtr in other thermoregulatory regions, such as the raphe nuclei, and the detailed mechanisms of regulation in these regions are not clear (15). In this study, we have shown that Oxtr expressed in the RMR has an important role in thermoregulation during cold conditions.

## Materials and Methods

### Animals

The care and use of mice in this study was approved by the Institutional Animal Care and Use Committees of Tohoku University and Jichi Medical University. *Oxtr*^−/−^ (5) mice, OXTR-Venus knock-in mice (31), and *Oxtr*-floxed mice (5) were generated as described previously. In brief, *Oxtr*^−/−^ mice are conventional knockout mice, which have deletion of exons 2 and 3 in *Oxtr* gene. In OXTR-Venus knock-in mice, the part of exon 3 of Oxtr gene is replaced with Venus gene. In *Oxtr*-floxed mice, exons 2 and 3 of *Oxtr* gene are surrounded by two loxP sites. The *Oxtr*^−/−^ mice used in this study were maintained on a mixed 129/Ola and C57BL/6J genetic background. Male mice were used in this study. Mice were kept at 25°C, and had standard chow diet and water *ad libitum*.

### Core Body Temperature Measurement

We measured the core body temperature using a rectal probe attached to a digital thermometer (Digi-Temper, Tsuruga Electric Works, Ltd.). The mice were held by the nape of the neck, and we inserted the rectal probe 1 cm deep in each time points. Mice were caged individually and fasted for 3 h (from 1000 to 1300) before placing them in a room maintained at 5°C (from 13:00) for the cold-exposure experiments. Mice were exposed cold condition for 6 h. During acute cold exposure, bedding was kept to a minimum. We prepared adipose tissue for hematoxylin and eosin (HE) staining and RNA extraction for RT-PCR, under these conditions.

### Hematoxylin and Eosin Staining

We fixed samples of interscapular BAT from 12-week-old mice in 4% paraformaldehyde solution, embedded them in paraffin, and sectioned them at a thickness of 5 μm. We then stained sections with HE.

### Quantitative Real-Time PCR Analysis

Total RNA was isolated from interscapular BAT obtained from 10- to 12-week-old male mice in each condition, and cDNA were produced from 1 μg of total RNA according to methods reported in a previous study (32). For real-time quantitative PCR, 1 μl of the cDNA product was added to 20-μl reaction volume containing 10 μl DyNAmo SyBR Green qPCR Kit (Finnzymes) and 1 μl of 12.5 pM primers (forward and reverse). For each sample, a parallel reaction was set up with acidic ribosomal phosphoprotein P0 (Arbp) as an endogenous control. The reactions were run in a DNA Engine Opticon System (MJ, Japan). Each reaction was performed in duplicate. In real-time PCR analysis, the following primers were used: UCP1, forward, 5′-GTGAAGGTCAGAATGCAAGC-3′ and reverse, 5′-AGGGCCCCCTTCATGAGGTC-3′; α2A-AR, forward, 5′-CGCTCAAAGCTCCCCAAAAC-3′ and reverse, 5′-GCTTCAGGTTGTACTCGATGGC-3′; β3-AR, forward, 5′-GGACCTGCACTACCACCTGT-3′ and reverse, 5′-CATGAGGCCTCTTCTTGGAG-3′; Arbp, forward, 5′-ATAACCCTGAAGTGCTCGACAT-3′ and reverse, 5′-GGGAAGGTGTACTCAGTCTCCA-3′.

### Cold Exposure of Mice for Immunostaining

Cold exposure of mice was performed in a climate chamber. Animals were placed individually in plastic cages with wire mesh lids and allowed access to food and water ad libitum. To acclimatize the animals to the climate chamber, they were placed in the chamber air-conditioned at 25°C for 2 h once per day (from 1,300 to 1,500 h), and this training was continued for 3 days. On the fourth day, they were exposed to a temperature of 5°C for 2 h (from 1300 to 1500 h). Control animals were kept at 25°C for 2 h on the fourth day.

### Immunohistochemistry

All mice were deeply anesthetized with avertin and perfused transcardially with saline (0.89% NaCl) followed by 4% paraformaldehyde in phosphate buffered saline (0.1M, PBS). The perfused brains were removed from the skull and postfixed overnight in 4% paraformaldehyde. The brains were then transferred to 30% sucrose at 4°C and frozen at −40°C. Coronal sections of 30 μm thickness were cut from the brain on a cryostat at −20°C.

Immunohistochemistry for Oxtr was performed using *Oxtr*^Venus/+^ mice. Sections were dipped in 0.5% H_2_O_2_ for 15 min and incubated with 10% normal goat serum (NGS) for 1 h. The sections were incubated overnight with anti-GFP rabbit antibody (MBL; 1:1000) in 0.3% Triton X-100 in PBS at room temperature. Sections were then dipped in 0.5% H_2_O_2_ for 20 min and washed in PBS three times for 5 min each. The sections were incubated overnight with biotinylated goat anti-rabbit IgG (Vector Lab; 1:1000) at 4°C, then incubated for 1 h at room temperature with avidin–biotin complex, and then with 3,3′-Diaminobenzidine solution.

For observation of Venus fluorescence, sections were incubated for 1 h with 10% NGS and then incubated overnight with anti-GFP rabbit antibody (MBL; 1:1000) in 5% NGS in PBS at 4°C. Sections were then incubated with Alexa 594-labeled goat anti-rabbit antibody (Molecular probes; 1:500) for 2 h at room temperature.

For double immunofluorescent detection of Venus and c-Fos, sections were incubated for 1 h with 10% normal donkey serum (NDS), and then incubated overnight with anti-c-Fos goat antibody (Santa Cruz; 1:250) in 5% ND and 0.3% Triton X-100 in PBS at 4°C. Sections were then incubated with Alexa 594-labeled donkey anti-goat antibody (1:500) for 2 h at room temperature. Following this, sections were labeled with anti-GFP to visualize Venus expression using the method as described above.

For double immunofluorescent detection of Venus and tryptophan hydroxylase 2 (TPH2), sections were incubated for 1 h with 10% NDS and then incubated overnight with anti-TPH2 sheep antibody (Millipore Bioscience Research reagents; 1:1000) in 5% NDS and 0.3% Triton X in PBS at 4°C. Sections were incubated with Alexa 594-labeled donkey anti-sheep antibody (1:500) for 2 h at room temperature. Next, these sections were incubated for 1 h with 10% NGS, and labeled with anti-GFP to visualize Venus expression using the method as described above. For double immunofluorescent staining of β-galactosidase (β-gal) and TPH2, anti-β-gal mouse monoclonal antibody (Promega; 1:300) and Alexa 488-labeled goat anti-mouse antibody (1:500) were used on the same protocol.

For double immunofluorescent detection of Venus and vGluT3, sections were incubated for 1 h with 10% NGS and then incubated overnight with anti-vGluT3 guinea pig antibody (provided by Kyoto university, Dr. Hioki; 1:100) in 5% NGS in PBS at 4°C. Sections were incubated with Alexa 594-labeled goat anti-guinea pig antibody (1:500) for 2 h at room temperature. Next, these sections were labeled with anti-GFP to visualize Venus expression using the method as described above. For double immunofluorescent staining of β-gal and vGluT3, anti-β-gal mouse monoclonal antibody (Promega; 1:300) and Alexa 488-labeled goat anti-mouse antibody (1:500) were used on the same protocol. All sections were washed three times in PBS and stained with DAPI. The sections were observed under a confocal laser-scanning microscope (FV1000, Olympus).

### Injection of AAV Vectors

All experiments using AAV vectors were performed at Animal Biosafety Level 1 (ABSL-1) rooms. A lab coat and gloves were worn when handling the virus. AAV vectors were prepared as described previously (15). 3.1 × 109 vg (virus genome)·μl^−1^ AAV vector, as described previously (15, 33, 34), was used in this study. One microliter of AAV-Oxtr or AAV-LacZ (control) vectors was injected into the RMR (stereotaxic coordinates: anteroposterior, −6.48 mm; mediolateral, ±0 mm; dorsoventral, +6 mm from Bregma) of *Oxtr*^−/−^ male mice. Similarly, 1 μl of AAV-Cre vector or AAV-LacZ vectors was injected into the RMR of *Oxtr*^fx/fx^ male mice. The mice were allowed to recover for at least 1 week before cold-exposure experiments.

### Statistical Analysis

Statistical analyses were performed using the statistical package SPSS for Windows (version 22, IBM, Armonk, NY, USA). Data are expressed as mean ± SEM. Body temperature was analyzed using two-way repeated analysis of variance (ANOVA) with the between-subject factor of group. Time point was used as the within-subject factor for the analysis of the body temperature data. Since overall ANOVA revealed that there was no difference in body temperature between *Oxtr*^−/−^ mice and AAV-LacZ injected *Oxtr*^−/−^ control mice, these data were combined as *Oxtr*^−/−^ group and used for body temperature analysis. Morphology of BAT and white adipose tissue (WAT) were analyzed using one-way ANOVA with the between-subject factor of group. Real-time PCR data were analyzed using two-way ANOVA with the between-subject factors of group and ambient temperature (25 vs. 5°C). Significant ANOVA results were followed by Tukey–Kramer *post hoc* comparisons. All alpha levels were set as 0.05.

## Results

### Oxtr was Expressed in the Rostral Medullary Raphe Nuclei

**Figure 1:**
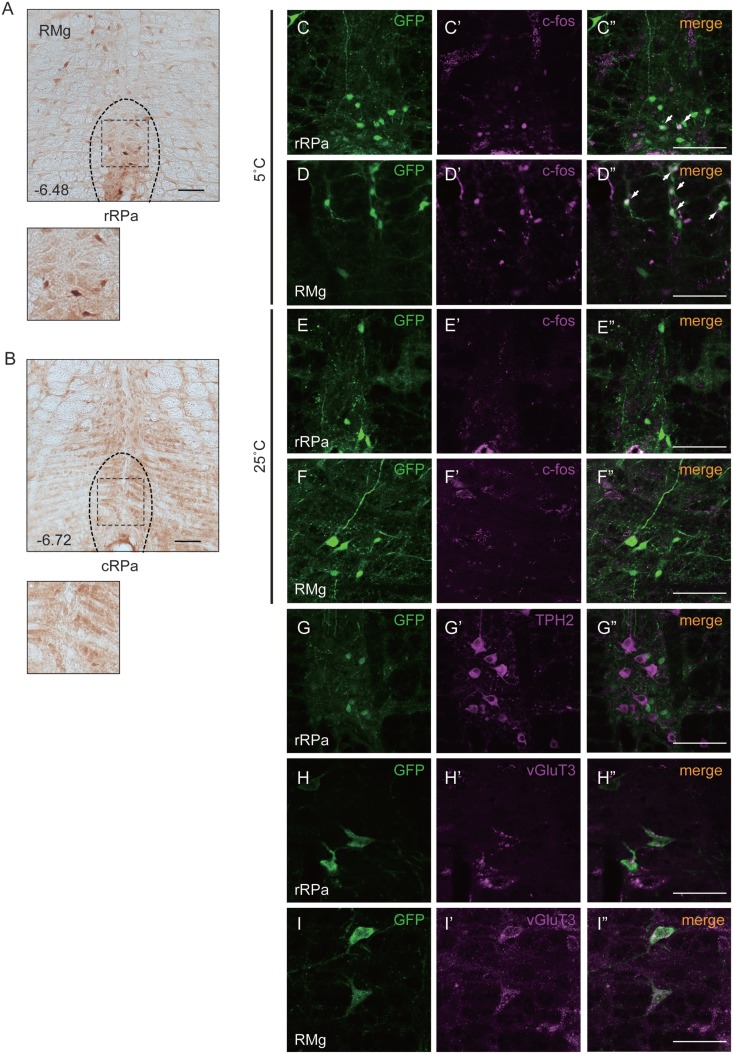
**Oxtr expressed neurons in the rostral raphe pallidus (rRPa) were activated by cold exposure**. **(A,B)** Cryosections of the RPa, indicating which of the Oxtr-Venus mice were immunostained with anti-GFP. Oxtr was mostly expressed in the rRPa **(A)**, but not in the caudal raphe pallidus (cRPa) **(B)**. Distance from bregma (millimeter) is indicated in the figure panel. **(C–F)** Oxtr-expressing neurons were activated during the cold condition, but not under normal conditions in the rRPa and RMG. Samples were immunostained with anti-GFP (green), anti-c-Fos (magenta), and merged. The arrows indicate neurons expressing both Venus and c-Fos. **(G–I)** Oxtr-expressing neurons were immunostained using both glutamatergic markers (vGluT3) and serotonergic markers (TPH2). Scale bar: 100 μm **(A–G)**, 50 μm **(H,I)**.

To investigate the function of Oxtr in thermogenesis, we focused on the RMR, a brain region that harbors pre-motor neurons regulating BAT. We confirmed the expression of Oxtr in the RMR by using OXTR-Venus knock-in mice. Oxtr was mostly expressed in the rostral RPa, but not in the caudal RPa (Figures 1A,B).

### Oxtr-Expressing Neurons were Activated upon Exposure to Cold

The expression pattern of Oxtr suggests that the Oxtr-expressing neurons in the RMR are important for thermogenesis during cold exposure. To elucidate this hypothesis, the activation of these neurons was analyzed by c-Fos immunostaining. Two different mouse groups were prepared for this experiment: one group was exposed to a temperature of 5°C and the other group was exposed to a temperature of 25°C. Only the group exposed to 5°C showed c-Fos expression in the RPa and RMg. During cold exposure, 29.9 ± 7.6% of Oxtr neurons in the RPa and 40.8 ± 17.4% of Oxtr neurons in the RMg were c-Fos positive (Figures 1C–F).

### Oxtr-Expressing Neurons in the RMR Showed Glutamatergic Properties

It is well known that the pre-motor neurons in the RMR can be classified into two kinds of groups: serotonergic neurons and vGluT3-positive glutamatergic neurons. Both serotonergic and vGluT3-positive neurons project to the T4 IML in the spinal cord and promote thermogenesis in BAT. To identify the properties of Oxtr-expressing neurons, we performed immunostaining for marker proteins of each type of neuron in the RMR obtained from OXTR-Venus mice. Although we had previously reported the co-expression of Oxtr and TPH2, a serotonergic neuronal marker, in the median raphe nucleus (MnR) and dorsal raphe (DR) nucleus (31), this co-expression was not observed in the RMR. Interestingly, expression of vGluT3, both in RPa and RMg, was strongly associated with Oxtr expression. 31.7 ± 11.1% of Oxtr neurons in the RPa and 66.5 ± 12.5% of Oxtr neurons in the RMg were expressed vGluT3 (Figures 1G–I).

### Loss of Thermogenesis in Oxtr-Deficient Mice was Rescued by RMR-Specific Oxtr Expression

**Figure 2:**
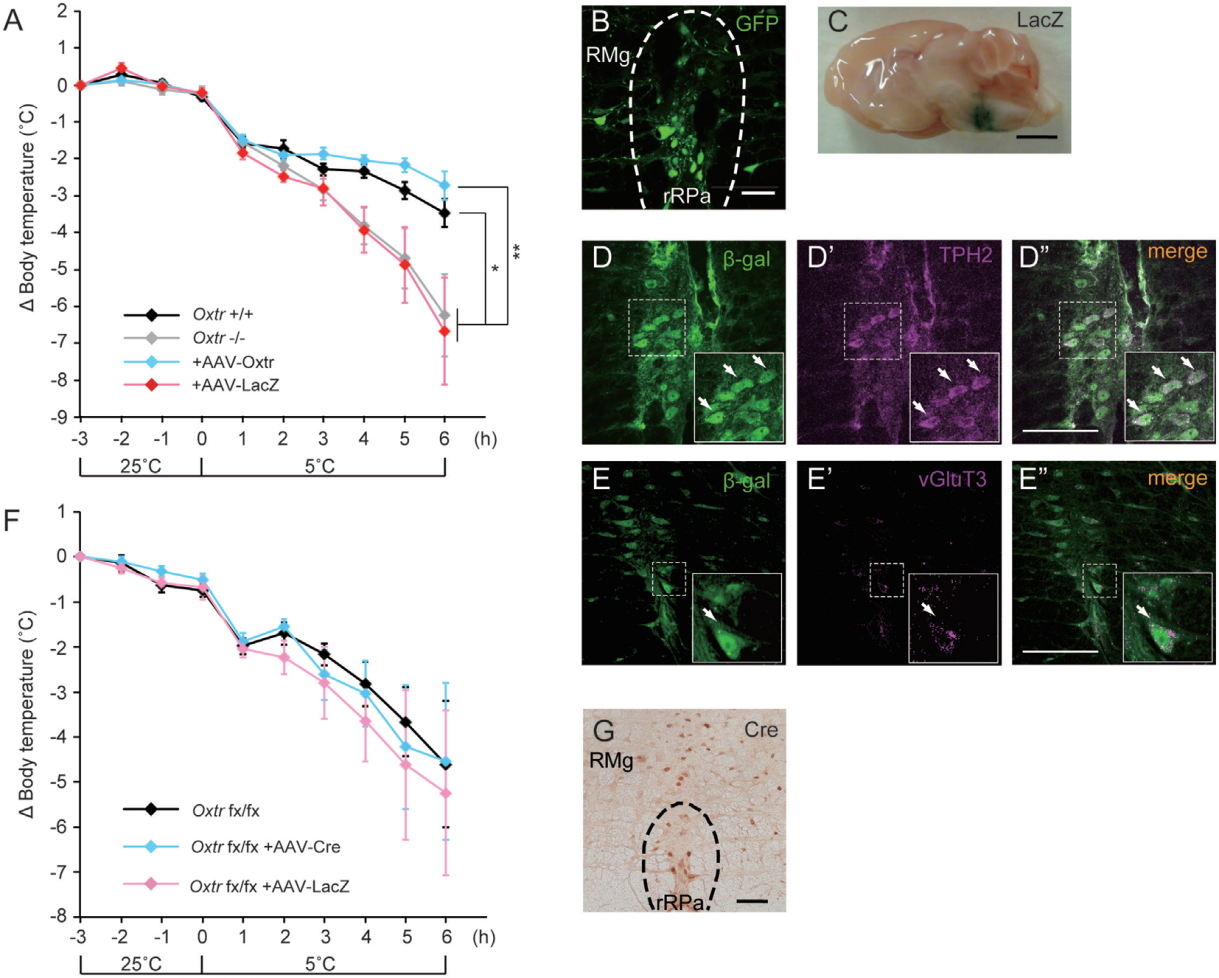
**Malfunction of thermoregulation in *Oxtr*-deficient animals was rescued by AAV-Oxtr injection**. **(A)** Core body temperature was measured in 10- to 13-week-old male *Oxtr*^+/+^ (*n* = 9), *Oxtr*^−/−^ (*n* = 8), +AAV-Oxtr (*n* = 8) and +AAV-LacZ (*n* = 8) mice. The data of *Oxtr*^−/−^ and +AAV-LacZ mice were treated as *Oxtr*-deficient group in this experiment. Dysfunction of body temperature control in *Oxtr*-deficient group was rescued by AAV-Oxtr injection into the RMR. **(B)** Immunohistochemical images with anti-GFP antibody on the rRPa of AAV-Oxtr-Venus-injected mice. **(C)** Injection site was visualized by LacZ-staining. **(D)** Double immunostaining of TPH2 and β-gal on the rRPa of AAV-LacZ injected mice. **(E)** Double immunostaining of vGlut3 and β-gal on the rRPa of AAV-LacZ injected mice. **(F)** Core body temperature was measured in 10- to 13-week-old male *Oxtr*^fx/fx^ (*n* = 5), +AAV-Cre (*n* = 5), and +AAV-LacZ (*n* = 5) mice. Region-specific deletion of Oxtr in the RMR did not cause any diminished thermoregulatory ability. **(G)** Immunohistochemical images with anti-Cre antibody on the rRPa of AAV-Cre injected mice. *p < 0.05, **p < 0.01, significant differences between groups. Scale bar: 50 μm **(B)**, 2 mm **(C)**, 100 μm **(D,E,G)**.

We had previously found that disruption of *Oxtr* diminishes thermoregulatory ability (13, 15). We therefore performed a region-specific rescue-of-expression experiment using an adeno-associated virus vector to verify this effect. An AAV-Oxtr vector was injected into the RMR of *Oxtr*^−/−^ mice (+AAV-Oxtr), and *Oxtr*^−/−^ mice injected with an AAV-LacZ vector were used as negative controls. Statistical analysis revealed that body temperature of +AAV-LacZ negative control mice was similar to that of *Oxtr*^−/−^ mice; therefore, both of the data were combined and treated as *Oxtr*^−/−^ group in the body temperature experiment. *Oxtr*^−/−^ group were less resistant to cold exposure when compared with *Oxtr*^+/+^ mice, in agreement with our previous finding. Interestingly, +AAV-Oxtr mice had higher body temperatures than *Oxtr*^−/−^ group after cold exposure (Figure 2A). This result suggests that RMR-specific Oxtr-expressing mice have robust thermoregulatory competence, and that RMR-specific Oxtr expression can recover the diminished thermoregulatory ability in *Oxtr*^−/−^ mice. AAV-Oxtr infection and expression in the RMR was confirmed (Figure 2B), though AAV vectors showed approximately 1 mm diameter of diffusion into surrounding brain regions, as was visualized by LacZ-staining (Figure 2C). We also evaluated the types of AAV-infected neurons by double immunostaining for β-gal and type-specific neuronal markers in AAV-LacZ mice. AAV vectors were then infected both in vGluT3-positive neurons and in TPH2-positive neurons (Figures 2D,E). We also analyzed the effect of region-specific deletion of Oxtr in the RMR using *Oxtr*^fx/fx^ mice injected with an AAV-Cre vector. RMR-specific deletion of Oxtr did not diminish thermoregulatory ability (Figure 2F), when Cre was expressed in the RMR (Figure 2G).

### The Morphology of BAT Cells was Rescued by RMR-Specific Oxtr Expression

**Figure 3:**
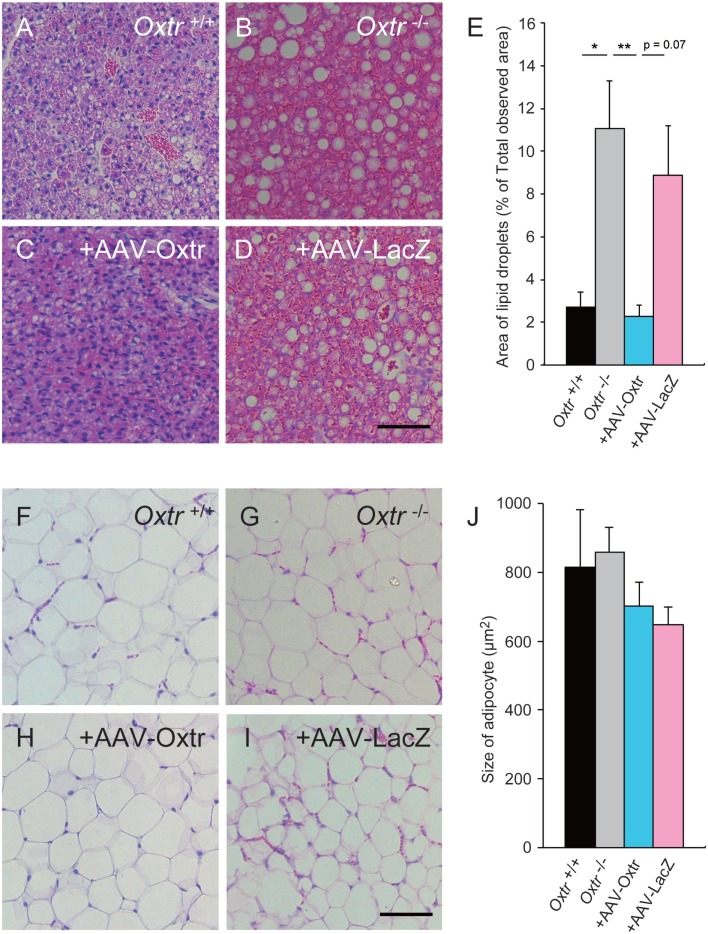
**Excessive lipid accumulation in the BAT of *Oxtr*^−/−^ mice was recovered by in AAV-Oxtr injection**. Hematoxylin and eosin stained brown **(A–D)** and white **(F–I)** adipose tissues from *Oxtr*^+/+^ mice **(A,F)**, *Oxtr*^−/−^ mice **(B,G)**, *Oxtr*^-/−^ mice with AAV-Oxtr injection into RMR **(C,H)** and *Oxtr*^−/−^ mice with AAV-LacZ injection into RMR **(D,I)**. **(E)** Areas of lipid droplets in BAT of *Oxtr*^+/+^ (*n* = 5), *Oxtr*^−/−^ (*n* = 7), +AAV-Oxtr (*n* = 6) and +AAV-LacZ (*n* = 6) mice. **(J)** Size of adipocytes in WAT of *Oxtr*^+/+^ (*n* = 4), *Oxtr*^−/^ (*n* = 5), +AAV-Oxtr (*n* = 5), and +AAV-LacZ (*n* = 6) mice. *; *p* < 0.05, ** *p* < 0.01, significant differences between groups. Scale bar: 60 μm.

In our previous study, *Oxtr*^−/−^ mice showed excessive lipid accumulation in BAT (13, 15). Recent studies have suggested that mice that show excessive lipid accumulation in BAT also have abnormal thermogenesis. We speculated that the recovery of thermogenesis in +AAV-Oxtr mice was associated with the recovery of BAT function. To investigate our speculation, we performed histological analysis on BAT obtained from these mice. The BAT obtained from cold exposed *Oxtr*^−/−^ mice showed excessive lipid accumulation when compared with *Oxtr*^+/+^ mice (Figures 3A,B). Interestingly, the sizes of lipid droplets in the BAT of +AAV-Oxtr mice were almost the same as those in the BAT of *Oxtr*^+/+^ mice (Figures 3C–E). This suggests that RMR-specific Oxtr expression recovers lipid metabolism. By contrast, the size of lipid droplets in WAT was unchanged in both +AAV-Oxtr and +AAV-LacZ. (Figures 3F–J).

### The Expression Profile of β-Adrenergic Receptors in BAT was not Rescued by RMR-Specific Oxtr Expression

**Figure 4:**
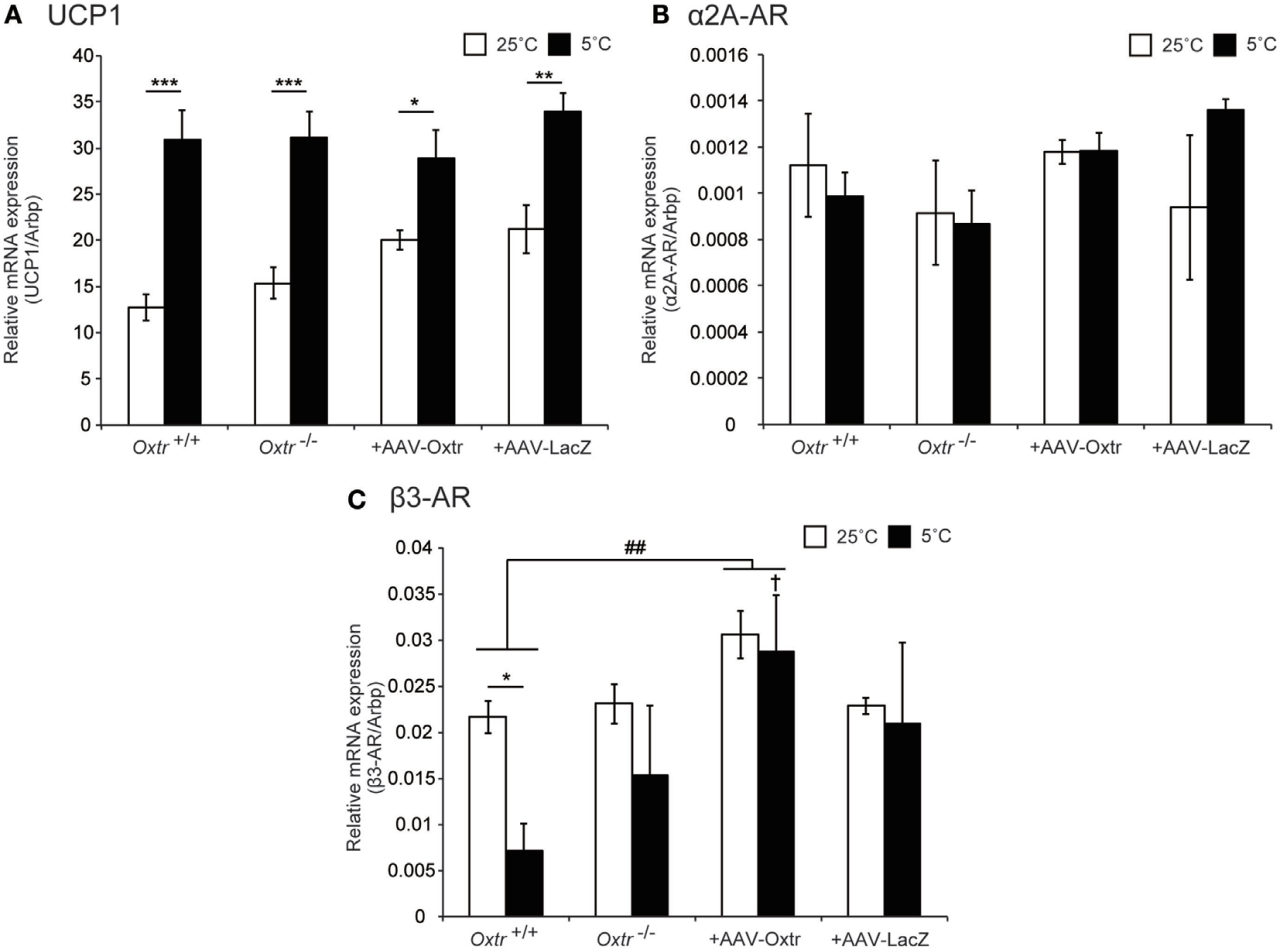
**Expression level of thermoregulation-related genes in BAT were quantified in *Oxtr*^+/+^, *Oxtr*^−/−^, +AAV-Oxtr, and +AAV-LacZ mice**. **(A)** UCP1, **(B)** α2A-AR, and **(C)** β3-AR expression were measured by quantitative PCR analysis. Mice used for the preparation of mRNA were treated in 25 and 5°C conditions (*n* = 5 for each groups and conditions). * *p* < 0.05, ** *p* < 0.01, *** *p* < 0.001, compared with 25°C condition; ^†^, *p* < 0.05, compared with *Oxtr*^+/+^ mice; ^##^, *p* < 0.01, significant difference between groups.

Brown adipose tissue is innervated by adrenergic sympathetic nerves, and expresses several types of adrenergic receptors (AR), including α2A-AR and β3-AR, which are considered to be important in thermogenesis (21). Previously, we reported that disruption of *Oxtr* causes an abnormal expression pattern of ARs in BAT (15). In this study, we performed quantitative PCR analysis to measure the expression of uncoupling protein 1 (UCP1, a key molecule involved in thermogenesis) and ARs to assess whether they were altered by RMR-specific Oxtr expression. The expression of UCP1 was elevated during cold exposure in both *Oxtr*^−/−^ and *Oxtr*^+/+^ mice. By contrast, the expression of α2A-AR remained unchanged, regardless of genotype and temperature conditions. Interestingly, the expression of β3-AR was significantly different between the normal temperature and cold conditions in *Oxtr*^+/+^ mice (Figures 4A–C). In RMR-specific Oxtr-expressing mice, both UCP1 and α2A-AR expression were not altered between +AAV-Oxtr and +AAV-LacZ mice. Intriguingly, the significant group difference was observed in the expression of β3-AR. β3-AR expression was significantly elevated in +AAV-Oxtr mice compared with that of *Oxtr*^+/+^ mice. Moreover, although β3-AR expression in *Oxtr*^+/+^ mice was down-regulated by cold exposure, down-regulation of β3-AR expression was not observed in other groups, including +AAV-Oxtr mice. The expression level of β3-AR in the BAT of *Oxtr*^+/+^ mice was significantly lower as compared with +AAV-Oxtr mice in the cold-exposure condition. The expression of β3-AR in +AAV-Oxtr mice was similar to that in +AAV-LacZ mice (Figures 4A–C).

## Discussion

The Oxt/Oxtr system plays a key role in the regulation of energy homeostasis. Previous studies have reported significant weight gain in male *Oxt*^−/−^ mice and *Oxtr*^−/−^ mice (13, 14). In addition, we observed that these mice exhibit defective thermoregulation under cold conditions (13–15). *Oxtr*^−/−^ mice also show an accumulation of fat in BAT, the principal organ that produces heat energy.

Reductions in temperature are detected by receptors located on the surface of the skin and in core body parts. This induces thermoregulatory circuits in the brain, causing noradrenalin to be released by SPNs and activating thermogenesis in BAT (27, 28). We predicted that the thermoregulatory defect in *Oxtr*^−/−^ mice was due to an irregularity in this thermoregulatory circuit, but the process was unknown. We recently reported that the Oxt/Oxtr system in the hypothalamus (dorsomedial hypothalamic nucleus and ventromedial hypothalamic nucleus; DMH/VMH) plays an important role in thermoregulation (15). The DMH/VMH are among the nuclei that compose the thermoregulatory circuit, and Oxtr is abundant in this region (28, 31). Under cold conditions, many neurons expressing Oxtr also expressed c-Fos (data not shown), and DMH/VMH-specific rescue of *Oxtr* by an AAV-Oxtr vector in *Oxtr*^−/−^ mice prevented the previously observed decrease in body temperature. These results suggest that Oxtr expressed in the DMH/VMH is important for thermoregulation (15). However, neurons expressing Oxtr are widely distributed in the brain and various thermoregulatory regions aside from the DMH/VMH (31). Therefore, this study specifically focused on the RMR and analyzed the thermoregulatory function of the Oxt/Oxtr system in this nucleus only.

Oxytocin receptor expression was observed in the RMR, which is generally known to regulate BAT activity. Interestingly, such expression was not observed in the caudal region of the medullary raphe (MR). Additionally, many reports have shown that c-Fos expression is increased in RMR neurons during cold conditions (25, 35). Accordingly, we observed that neurons expressing Oxtr in the RMR were c-Fos-positive. These results suggest that neurons expressing Oxtr are activated in cold conditions and may modulate thermoregulation. We therefore injected an AAV-Oxtr vector into the RMR of *Oxtr*^−/−^ mice with and measured their fasting-state body temperature during cold conditions. *Oxtr*^−/−^ animals showed significantly lower body temperatures compared with *Oxtr*^+/+^ mice 3 h after cold exposure, regardless of immediate sympathetic nerve activation in this condition. This paradoxical phenomenon might be affected by both lower sympathetic tone and dysfunction of the Oxt/Oxtr system (36). Depletion of FFA in *Oxtr*^−/−^ mice resulted from the fasting condition and a decreased capacity for fat breakdown (15). Food intake is also an important factor in body temperature control: *Oxtr*^−/−^ mice show body temperature disregulation in cold environments not only in fasting states (15) but also in fed states (13). The amount of food intake during cold exposure was similar between *Oxtr*^+/+^ mice and *Oxtr*^−/−^ mice (13). In order to exclude the effect of food on thermoregulation, a fasting condition of longer than 3 h might have been more appropriate for this study. However, since *Oxtr*^+/+^ mice had very low core temperatures at the end of the 6-h period, fasting during the experiment could have affected adequate thermoregulation.

+AAV-Oxtr mice had higher body temperatures during the cold condition when compared with *Oxtr*^−/−^animals, which suggests that the Oxt/Oxtr system in the RMR is an important component of the thermoregulatory system. However, the Oxt/Oxtr system is also important for stress responses (37) and stress induces thermogenesis (38). Because we measured the body temperature of mice using a probe, measurement-induced stress may have affected the body temperature of these mice. We also exposed AAV-Cre vector-injected *Oxtr*^fx/fx^ mice to cold conditions, and these mice showed thermoregulatory ability similar to that of *Oxtr*^+/+^ mice. Based on this result, we speculated that the Oxt/Oxtr system in the RMR is just one of the factors involved in regulating thermogenesis in cold conditions, and defects in the function of this system in the RMR can be compensated for by other major functions. Although shivering behavior in mice across all groups appeared normal, a detailed analysis of this behavior may be more informative.

Anatomical studies have indicated that vGluT3-expressing glutamate neurons and serotonin neurons projecting from the RMR to the IML influence the activation of BAT SPNs (25, 28, 30). Co-expression of Oxtr and vGluT3 was observed in several neurons in the RMR, whereas Oxtr and TPH2 co-expression in neurons was hardly detected. Approximately 60–70% of vGluT3-positive neurons expressed c-Fos during cold exposure (25). These results suggested that the Oxt/Oxtr system in the RMR may regulate the release of glutamate; however the exact relationship is still unknown and open to future study.

We found an excessive accumulation of lipid droplets in the BAT of *Oxtr*^−/−^ mice and, therefore, expected these mice to have abnormal fat metabolism (13, 15). We collected WAT and BAT from *Oxtr*^+/+^, *Oxtr*^−/−^, +AAV-Oxtr, and +AAV-LacZ mice that had been exposed to cold conditions for 6 h, and observed their morphology using HE staining. We found that +AAV-Oxtr mice showed scarce accumulation of lipid droplets in BAT when compared with *Oxtr*^−/−^ mice. By contrast, there were no changes in WAT morphology in +AAV-Oxtr mice (Figures 3C,D). Taken together, these results show that injection of the AAV-Oxtr vector prevented the accumulation of lipid droplets in the BAT of *Oxtr*^−/−^ mice, and caused them to recover their thermoregulatory ability.

Finally, we analyzed the expression of major heat production proteins in the BAT of *Oxtr*^+/+^, *Oxtr*^−/−^, +AAV-Oxtr, and +AAV-LacZ mice using quantitative RT-PCR. We were particularly interested in the expression of UCP1, α2A-AR, and β3-AR, since UCP1 is major heat production molecule in BAT, and our previous study reported aberrant expression of α2A-AR and β3-AR in *Oxtr*^−/−^ mice when compared with WT mice (15). α2A-AR suppresses, and β3-AR promotes heat production in BAT. There was no difference in the expression of UCP1 between groups in both normal and cold conditions, and UCP1 was significantly induced by cold exposure in both genotypes. Previous reports have shown that obese animals exhibit aberrant UCP1 expression in BAT (21, 39, 40). Contrary to these findings, *Oxtr*^−/−^ mice exhibited obesity (13) with normal UCP1 expression; however, thermoregulatory ability was nonetheless compromised. The uncoupling activation of UCP1 is activated by long-chain fatty acids (21). Although UCP1 activation may be normal in the BAT of *Oxtr*^−/−^ mice, its activity may be diminished due to defected lipolysis (15). The expression level of α2A-AR was similar across all mice groups and in both temperature conditions. By contrast, the expression of β3-AR significantly varied by conditions. Several previous studies have reported a dramatic down-regulation of β3-AR gene expression upon adrenergic stimulation of BAT, both *in situ* and in culture (41). We observed decreased expression of the β3-AR gene in the BAT of *Oxtr*^+/+^ mice; however, this effect was diminished in *Oxtr*^−/−^ mice, indicating abnormal noradrenergic signal input in *Oxtr*^−/−^ mice. Although body temperature control was recovered and the amount of lipid droplets in BAT during cold conditions was reduced in +AAV-Oxtr mice, a high level of β3-AR expression was nonetheless observed in the cold condition. The expression of β3-AR in +AAV-Oxtr mice was also higher than in *Oxtr*^+/+^ mice. This higher level of β3-AR expression in +AAV-Oxtr mice may contribute to the recovery of body temperature control, through the promotion of fatty acid release accompanied by the activation of the potentiated uncoupling property of UCP1. However, recovery of noradrenergic input in these mice may still be insufficient.

The AAV-Oxtr vector is not neuron specific. It is therefore possible that +AAV-Oxtr mice have more Oxtr protein in the RMR than *Oxtr*^+/+^ mice, especially since we observed diffused infection of AAV around the RMR. The overexpression of Oxtr in this region may have affected the result of our experiment. In addition, AAV vectors infected not only vGluT3-positive neurons, but TPH2-positive neurons as well. In a previous study, we reported co-expression of Oxtr and TPH2 in the MnR and DR, and showed that Oxtr facilitated serotonin release in the MnR, (31). Therefore, injection of the AAV-Oxtr vector into *Oxtr*^−/−^ mice may result in the promotion of serotonin neurons in the RMR. Since serotonin also plays an important role in body temperature regulation (42, 43) and the expression of ARs in adipose tissues (44), putative alteration of serotonergic function in +AAV-Oxtr mice may have affected our results.

On the other hand, our previous study reported that *Oxtr*^−/−^ mice showed increased expression of α2A-AR and decreased expression of β3-AR in BAT (15). Because Oxtr is not expressed in mature brown adipocytes, we expected that the abnormal expression of ARs would be due to defective noradrenergic input from SPNs. However, we found no abnormality in the expression of α2A-AR, and only β3-AR expression was altered in the cold condition in this study. Different complicated results were observed between in the previous study and in the present study. In the previous study, we prepared tissue from satiated mice (15), but this experiment was carried out under fasting conditions. This difference is especially relevant as it has been reported that β3-AR mRNA expression pattern in BAT varies between fed and fasted conditions. The expression of β3-AR in BAT is approximately three times greater in the fasted condition when compared with the fed condition (45). In addition, our previous study involved exposing mice to cold conditions for 2 h, but the present study entailed exposure to cold conditions for a period of 6 h. These methodological differences may account for differences in results between the present and previous study.

In summary, our findings suggest that the Oxt/Oxtr system in the RMR is an important regulator of thermoregulation in mammals exposed to cold conditions. We have also discovered a thermoregulatory function of the Oxt/Oxtr system in the DMH/VMH (15), and this system may have thermoregulatory roles in other regions, including peripheral tissue. Previous studies have shown that the Oxt/Oxtr system is necessary for the regulation of obesity and thermoregulatory ability, and we have elucidated a part of these functions. Our findings may also be applied in the modulation of BAT activity for obesity treatment, which is known to be a significant global health concern.

## Author Contributions

YT, YK, YH, and AO performed this research. YT, YK, YH, SH, and KN designed this research. HM, KO, and KS contributed essential reagents of this research. YT and YK analyzed the data. YK, YH, and KN wrote the paper.

## Conflict of Interest Statement

The authors declare that the research was conducted in the absence of any commercial or financial relationships that could be construed as a potential conflict of interest.
